# Supplementary material for: Humidity-dependent lubrication of highly loaded contacts by graphite and a structural transition to turbostratic carbon
Source: Nat Commun. 2022 Oct 10;13:5958. doi: 10.1038/s41467-022-33481-9 (PMC9550797; doi:10.1038/s41467-022-33481-9)
Supplement: Supplementary file 1 — Supplementary Information [file 41467_2022_33481_MOESM1_ESM.pdf]

Supplementary Information

Humidity-dependent lubrication of highly loaded contacts by graphite and a structural transition to turbostratic carbon

Carina Elisabeth Morstein[1,2,†], Andreas Klemenz[2,†], Martin Dienwiebel[1,2,*], and Michael Moseler[2,3,4,5,*]

[1]*Karlsruhe Institute of Technology (KIT), IAM - Institute for Applied Materials, MicroTribology Center µTC, Straße am Forum 7, 76131 Karlsruhe, Germany*
[2]*Fraunhofer-Institute for Mechanics of Materials IWM, MicroTribology Center µTC, Wöhlerstraße 11, 79108 Freiburg, Germany*
[3]*Institute of Physics, University of Freiburg, Hermann-Herder-Straße 3, 79104 Freiburg, Germany*
[4]*Freiburg Materials Research Center, University of Freiburg, Stefan-Meier-Str. 21, 79104 Freiburg, Germany*
[5]*Cluster of Excellence livMatS, Freiburg Center for Interactive Materials and Bioinspired Technologies, University of Freiburg, Georges-Köhler-Allee 105, 79110 Freiburg, Germany*
[†]*These authors contributed equally to this work.*
[*]*Corresponding authors. E-mails: martin.dienwiebel@kit.edu, michael.moseler@iwm.fraunhofer.de.*

September 19, 2022

Supplementary Information

Estimation of the Capillary Forces

Previous investigations by Arif *et al.* explain the high wear at high humidity by the formation of capillary necks and bridges [1, 2], causing massive adhesion between the pin and the graphite coating. To examine this possibility, the capillary force F_c for our experiments was calculated according to Equation 1 [3].

$$F_c = 4\pi\gamma R \cos\theta \tag{1}$$

Where γ is the surface tension (for water circa $72.8 \cdot 10^{-3}\,\mathrm{N\,m^{-1}}$ at 25 °C), R is the radius of the counterbody (here $1 \cdot 10^{-3}\,\mathrm{m}$, and θ is the contact angle. Assuming a mildly hydrophilic surface and consequently a contact angle of 60° [4], the resulting capillary force was calculated to be 0.46 mN. Hence, this accounts only for 0.1% of the force applied onto the coating. Even for a higher hydrophilicity with a contact angle of θ = 10° the resulting capillary force would not be drastically higher (only 0.90 mN). Thus, capillary forces can be ruled out regarding the cause of the observed high wear at high humidity values.

Estimation of Flash Temperature

Supplementary Table 1: Variables for the calculation of the flash temperature.

Variable	Value
λ	$50\,\mathrm{W\,m^{-1}\,K}$
ρ	$7850\,\mathrm{kg/m^3}$
c	$500\,\mathrm{J\,kg^{-1}\,K}$
v	$0.5\,\mathrm{mm\,s^{-1}}$
r (50 MPa)	$7.17 \cdot 10^{-3}\,\mathrm{m}$
r (1 GPa)	$1.38 \cdot 10^{-5}\,\mathrm{m}$
μ (50 MPa)	0.28
μ (1 GPa)	0.14
F_N (50 MPa)	5.6 mN
F_N (1 GPa)	402 mN

For the estimation of the flash temperature we used the formulas by Blok and Jaeger [5]. As a first step, we had to ensure that the Peclet number was lower than 1 to use the quasi-static approach. The heat conductivity χ is calculated with the heat conductivity coefficient λ, the density ρ, and the specific heat capacity c, see Equation 2.
With this, the Peclet number Pe was calculated together with the experimental velocity v and the Hertzian radius r. For both normal forces F_N and hence Hertzian radii, the Peclet number is smaller than 1, thus the quasi-static approach from Blok and Jaeger could be used for the calculation of the flash temperature θ (see Equation 4).

$$\chi = \frac{\lambda}{\rho c} \tag{2}$$

$$Pe = \frac{vr}{2\chi} \tag{3}$$

$$\theta = \frac{1}{8} \times \frac{\mu v F_N}{\lambda r} \tag{4}$$

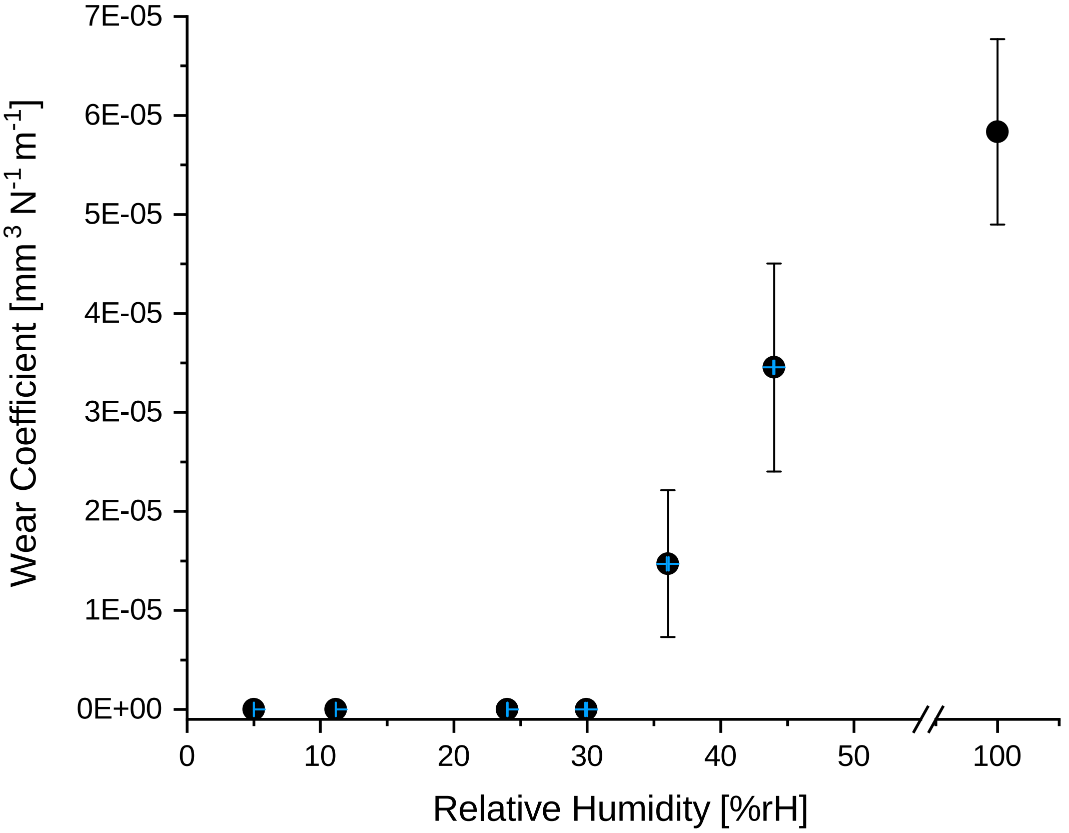

Supplementary Figure 1: **Wear coefficient of the counterbodies in comparison to the respective humidity.** The wear coefficient is calculated as stated in the experimental section of the main manuscript. For the experiments conducted from ≤ 5 to $30\,\%\,\mathrm{RH}$ no wear was detected, thus the wear coefficient is zero. The error bars represent the standard error of the mean of all measurements.

Sliding Simulations with Perpendicular Loops

In Fig. 9 and 10 of the main text, sliding of two identically oriented graphite crystals (both consisting of graphene planes perpendicular to the sliding direction) are presented. In these figures, the initial sliding interface is given by parallel loops terminating the upper and lower crystal (see inset in Supplementary Fig. 7c). To gain insight into the sliding behavior of different loop orientations, we simulate the same system with the upper crystal rotated by 90° (see inset in Supplementary Fig. 7f). Here, the $\langle 0001 \rangle$-direction of the lower crystal and the $\langle 12\bar{3}0 \rangle$-direction of the upper crystal are oriented

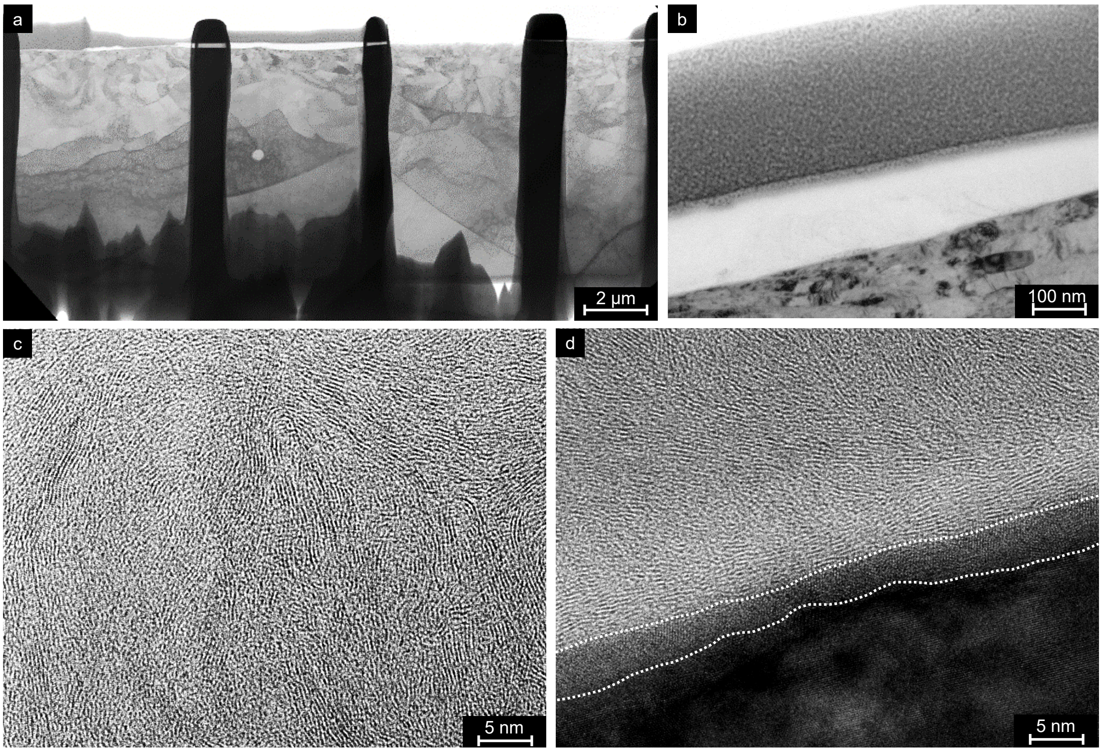

Supplementary Figure 2: **Transmission Electron Microscopy images.** Bright-Field (a, b) and High Resolution Transmission Electron Microscopy (c, d) images of the lamella prepared after the experiment at 1 GPa and $\leq$ 5 % RH.

in sliding direction, so the graphene layers in the two crystals as well as the terminating loops are oriented perpendicular to each other. The system displayed in Supplementary Fig. 7d consist of 384 carbon atoms with a cross-sectional area of $12.87 \times 12.87\,\text{Å}^2$. These dimensions are chosen to minimize the mismatch due to the different orientations of the crystals. The other simulation parameters correspond to those of the systems with identically oriented crystals described in the main text. To investigate the influence of humidity, dry systems and systems with 1-16 water molecules between the surfaces are simulated. For 0-4 water molecules these are repeated 4 times, while systems with 8-16 water molecules are simulated only once.

The results presented in Supplementary Fig. 7 indicate a lower reactivity of pairings of perpendicular loops. At a normal pressure of 1 GPa, no cold welding of the surfaces is observed, even in dry system (Supplementary Fig. 7d). At pressures of 3 and 5 GPa (Supplementary Fig. 7e,f), the behavior is similar to that observed for parallel-aligned loops (Supplementary Fig. 7b,c). Small amounts of water predominantly lead to cold welding of the surfaces at comparable shear stresses. However, in some cases it takes a certain time after the onset of sliding for cold welding to occur, as indicated by the

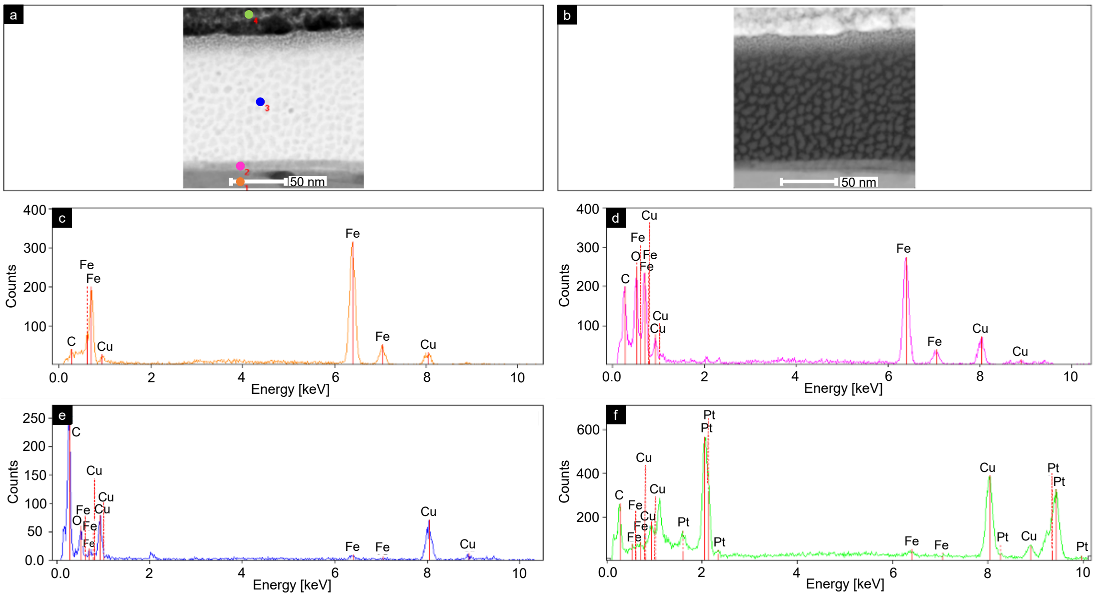

Supplementary Figure 3: **Energy Dispersive X-Ray Spectroscopy.** Measured after the experiment at 1 GPa and $\leq$ 5 % RH at different regions. (a,b) TEM images recorded in bright field and dark field mode at the region of interest. c) underlying iron substrate, d) newly formed iron carbide layer, e) newly formed carbon layer, and f) protective Platinum layer.

data points for water films at 1-4 water molecules in Supplementary Fig. 7e. For larger amounts of water, water films form between the surfaces, and the shear stresses in these cases are slightly lower than for parallel graphene layers. The transitions between the cold welding and water film regimes occur at slightly lower water densities for perpendicular graphene loops. Repassivation of the cold welded perpendicular loops is not observed on the time scale of the simulation suggesting a reduced tendency for t-C formation.

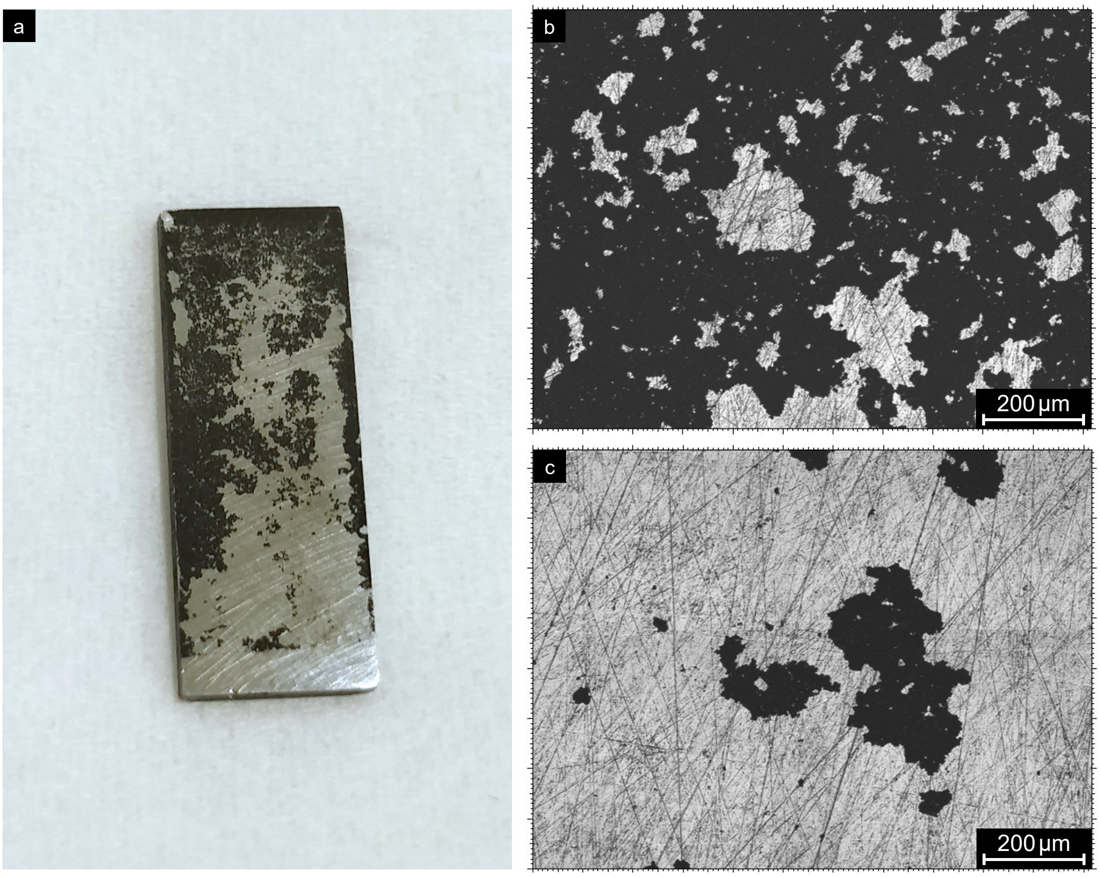

Supplementary Figure 4: **Photographic and microscopic images of an graphite-coated iron plate after the immersion experiment.** (a) Photography of an graphite coated iron plate was immersed in water and put into an ultrasonication bath for 1 min. (b and c) confocal microscopy images of two randomly selected regions on the plate. As one can see, the majority of the coating was swept away as it detached easily from the substrate.

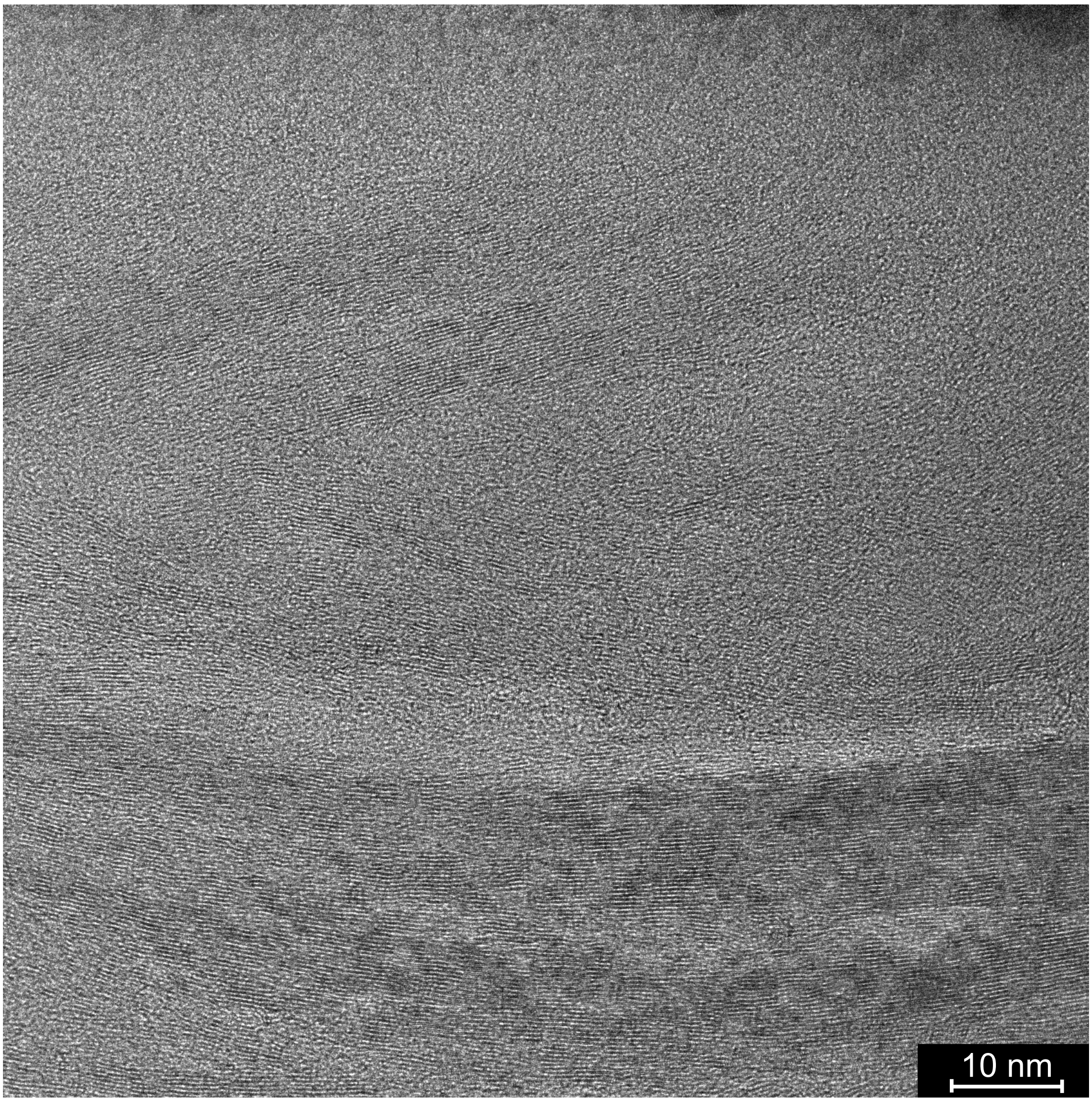

Supplementary Figure 5: **High-resolution TEM image of the inset of Fig. 5c.** Visible on the bottom of the image are the highly ordered graphene bundles which are transitioned into turbostratic carbon (t-C) at the sliding interface on the upper region of the image.

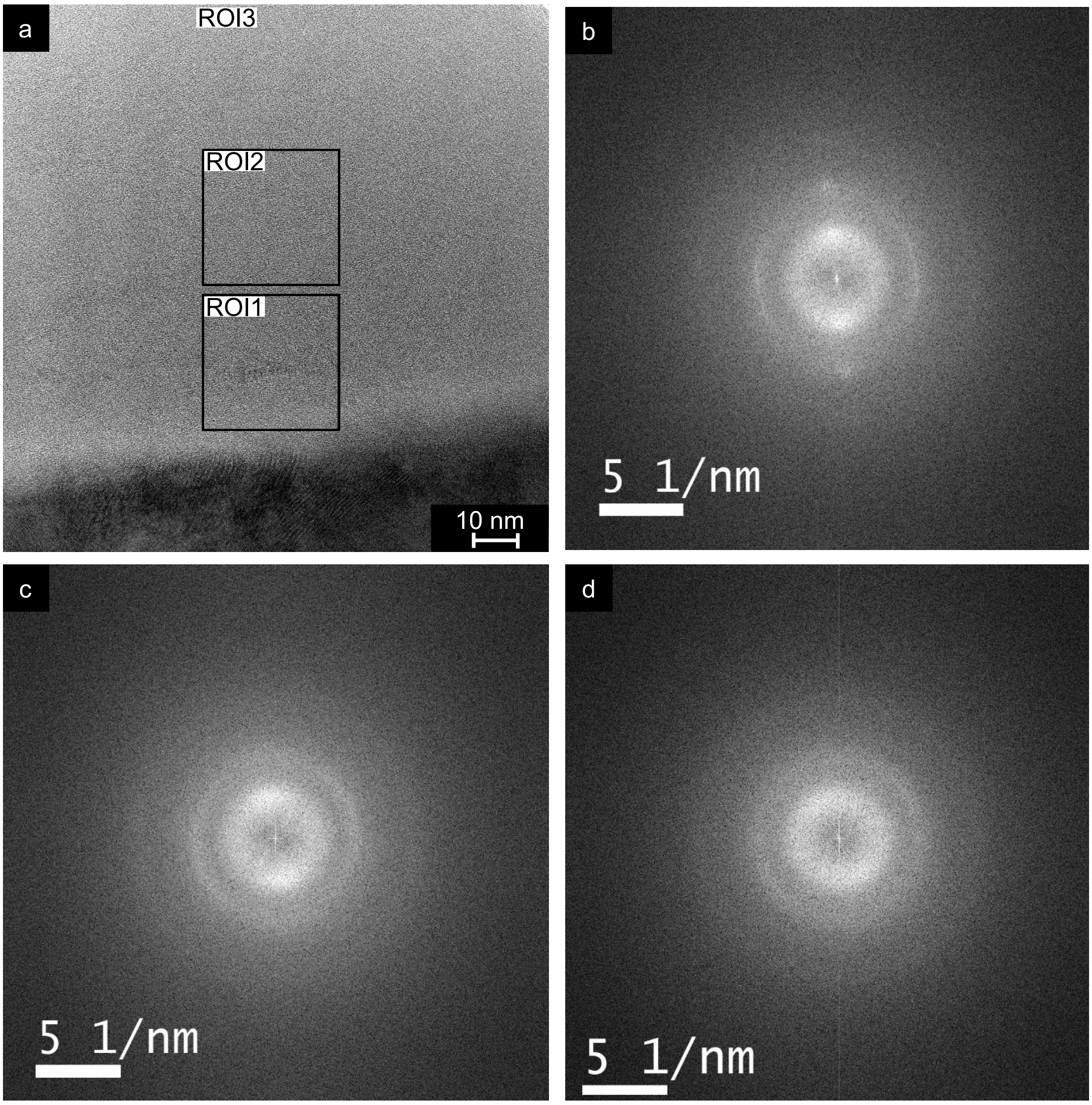

Supplementary Figure 6: **Fast Fourier Transformation (FFT) of the HR-TEM picture taken after the experiment at** 1 GPa **and** $\leq$ 5 % RH**.** (a) HR-TEM image with marked regions of interest (ROIs), (b) ROI1 close to substrate, (c) ROI2 in the middle, and (d) ROI3 close to sliding interface. The halos become more diffused when going from the substrate to the sliding interface, representing the transformation from polycrystalline graphite to t-C and its gradient when going from the substrate towards the sliding interface.

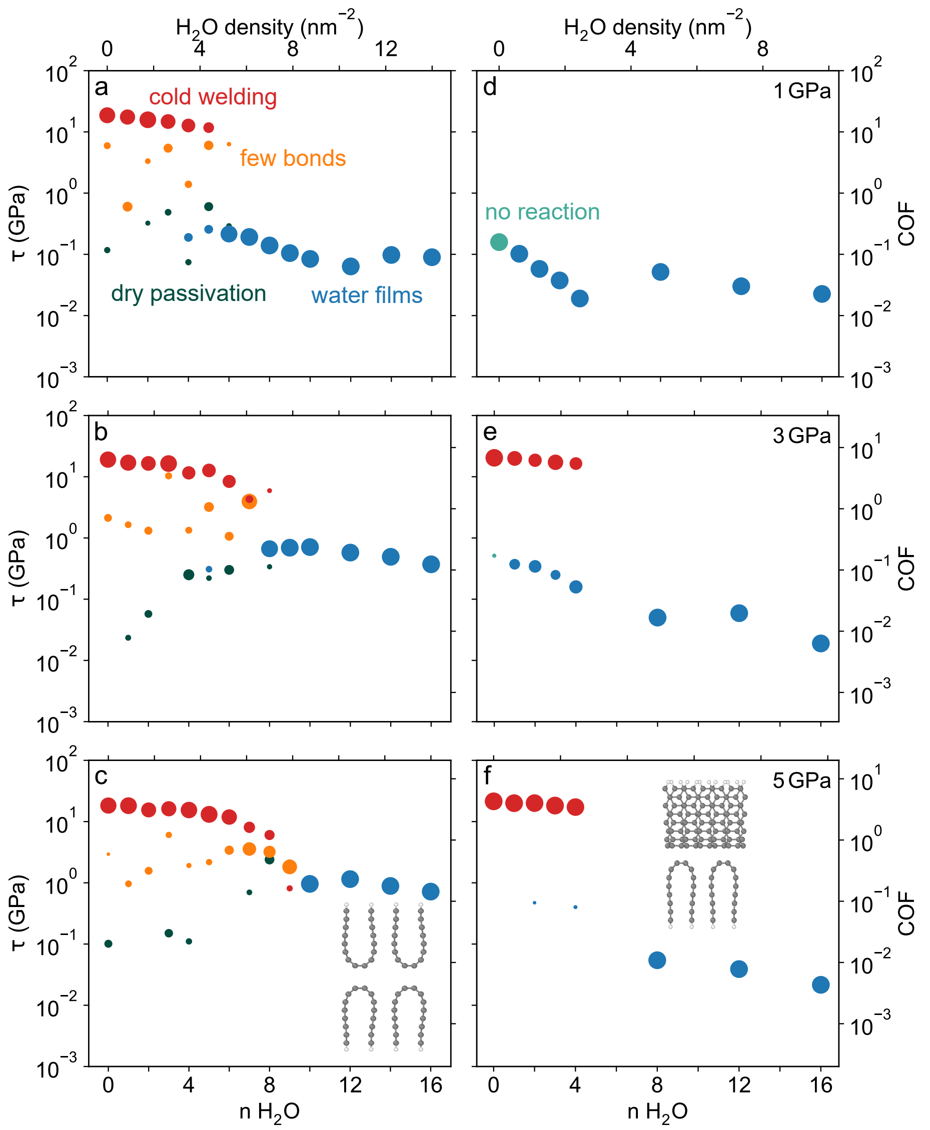

Supplementary Figure 7: **Typical shear stresses in the different regimes for parallel and perpendicular orientation of the graphene layers to each other.** The steady state shear stresses τ of the sliding simulations with parallel (a,b,c) and perpendicular loop orientation (d,e,f) on the upper and lower graphite crystal are compared (see insets in panel c and f for the start configurations of the dry systems, respectively). Panels a-c are essentially the data shown in Fig. 10b for 0-16 water molecules, while panels d-f provide additional insights into the sliding regimes for non-aligned loops. In both columns, two main regimes are observed: cold welding (red discs) and water lubrication (blue discs). Interestingly, perpendicular loops seem to be more stable (requiring higher pressures for cold welding and the transition to the water lubrication regime sets in for smaller water densities). For perpendicular loops, repassivation (yellow and olive discs) after cold welding is not observed on the timescale of our simulations.

Supplementary References

[1] Arif, T., Colas, G. & Filleter, T. Effect of humidity and water intercalation on the tribological behavior of graphene and graphene oxide. *ACS Appl. Mater. Interfaces* **10**, 22537–22544 (2018).

[2] Arif, T., Wang, G., Sodhi, R. N. S., Colas, G. & Filleter, T. Role of chemical vs. physical interfacial interaction and adsorbed water on the tribology of ultrathin 2D-material/steel interfaces. *Tribol. Int.* **163**, 107194 (2021).

[3] Butt, H.-J. & Kappl, M. *Surface And Interfacial Forces, 2nd ed* (Wiley-VCH, Weinheim, Germany, 2018).

[4] Kozbial, A., Trouba, C., Liu, H. & Li, L. Characterization of the intrinsic water wettability of graphite using contact angle measurements: Effect of defects on static and dynamic contact angles. *Langmuir* **33**, 959–967 (2017).

[5] Rowe, K. G., Bennett, A. I., Krick, B. A. & Sawyer, W. G. In situ thermal measurements of sliding contacts. *Tribol. Int.* **62**, 208–214 (2013).
