# Supplementary material for: Humidity-dependent lubrication of highly loaded contacts by graphite and a structural transition to turbostratic carbon
Source: Nat Commun. 2022 Oct 10;13:5958. doi: 10.1038/s41467-022-33481-9 (PMC9550797; doi:10.1038/s41467-022-33481-9)
Supplement: Supplementary file 3 — Description of Additional Supplementary Files [file 41467_2022_33481_MOESM3_ESM.pdf]

Description of Additional Supplementary Files

File Name: Supplementary Movie 1
Description: Video of the trajectory from which the snapshot in Fig. 8a was taken.

File Name: Supplementary Movie 2
Description: Video of the trajectory from which the snapshot in Fig. 8c was taken.

File Name: Supplementary Movie 3
Description: Video of the trajectory from which the snapshot in Fig. 8e was taken.

File Name: Supplementary Movie 4
Description: Video of the trajectory from which the snapshot in Fig. 8g was taken.

File Name: Supplementary Movie 5
Description: Video of the trajectory from which the snapshots in Fig. 9a-d were taken and from which the shear stresses in Fig. 9e were obtained.

File Name: Supplementary Movie 6
Description: Video of the trajectory from which the snapshots in Fig. 9f-i were taken and from which the shear stresses in Fig. 9j were obtained.

File Name: Supplementary Movie 7
Description: Video of the trajectory from which the snapshots in Fig. 9k-n were taken and from which the shear stresses in Fig. 9o were obtained.
